# Diarrhoeal pathogens in the stools of children living with HIV in Ibadan, Nigeria

**DOI:** 10.3389/fcimb.2023.1108923

**Published:** 2023-03-13

**Authors:** Oyeniyi S. Bejide, Mariam A. Odebode, Babatunde O. Ogunbosi, Olukemi Adekanmbi, Kolawole O. Akande, Temitope Ilori, Veronica O. Ogunleye, Victoria U. Nwachukwu, Aghogho Grey-Areben, Elizabeth T. Akande, Iruka N. Okeke

**Affiliations:** ^1^ Department of Microbiology, Chrisland University, Abeokuta, Ogun State, Nigeria; ^2^ Department of Pharmaceutical Microbiology, University of Ibadan, Ibadan, Oyo State, Nigeria; ^3^ Department of Paediatrics, College of Medicine, University of Ibadan, Ibadan, Oyo State, Nigeria; ^4^ Department of Medicine, College of Medicine, University of Ibadan, Ibadan, Oyo State, Nigeria; ^5^ Department of Community Medicine, College of Medicine, University of Ibadan, Ibadan, Oyo State, Nigeria; ^6^ Department of Medical Microbiology & Parasitology, University College Hospital, Ibadan, Oyo State, Nigeria

**Keywords:** diarrhoea, human immunodeficiency virus, diarrhoeagenic Escherichia coli, enteroaggregative Escherichia coli, enteric pathogens, faecal occult blood, Nigeria

## Abstract

**Introduction:**

Diarrhoea can be debilitating in young children. Few aetiological investigations in Africans living with human immunodeficiency virus (HIV) have been performed since antiretrovirals became widely available.

**Methods:**

Stool specimens from children with diarrhoea living with HIV, and HIV-uninfected controls, recruited at two hospitals in Ibadan, Nigeria, were screened for parasites and occult blood, and cultured for bacteria. Following biochemical identification of at least five colonies per specimen, diarrhoeagenic Escherichia coli and Salmonella were confirmed by PCR. Data were line-listed and comparisons were made using Fisher’s Exact test.

**Results:**

Only 10 children living with HIV could be enrolled during the 25-month study period and 55 HIV-uninfected children with diarrhoea were included for comparison. The most common pathogens overall were enteroaggregative E. coli (18/65, 27.7%), enteroinvasive E. coli (10/65, 15.4%), Cryptosporidium parvum (8/65, 12.3%) and Cyclospora cayetanensis (7/65, 10.8%). At least one pathogen was detected from seven of ten children living with HIV and 27 (49.1%) HIV-uninfected children. Parasite detection was associated with HIV positive status (p=0.03) with C. parvum specifically recovered more commonly from children living with HIV (p=0.01). Bacterial-parasite pathogen combinations were detected in specimens from four of ten children living with HIV but only 3(5.5%) HIV-uninfected children (p=0.009). Stools from five of ten children living with HIV and 7(12.7%) HIV-negative children (p = 0.014) contained occult blood.

**Discussion:**

Even though children living with HIV present infrequently to Ibadan health facilities with diarrhoea, their greater propensity for mixed and potentially invasive infections justifies prioritizing laboratory diagnosis of their stools.

## Introduction

Diarrhoea is the passage of three or more loose stools per day and can lead to appetite loss, dehydration and subsequent weight loss (Jensen et al., 2014). The incidence of diarrhoea is particularly high in low-income settings where sanitary conditions are largely inadequate, of which there are many in Africa (Botero et al., 2003). Unfortunately, many of these areas also have a high proportion of people living with human immunodeficiency virus (HIV) who, when immunocompromised, are susceptible to a plethora of opportunistic infections that can result in diarrhoea (Nwosu et al., 2014). Deaths from diarrhoea are declining but children under five in Africa may have up to an annual average of twelve episodes which places them at risk of death, malnutrition and significantly affects their well-being (Okeke, 2009; Farthing et al., 2013; Guarino et al., 2014; Zollner-Schwetz and Krause, 2015; Bruzzese et al., 2018).

Infectious diarrhoea can be caused by many viral, bacterial and parasitic agents. These include but are not limited to *Salmonella* and diarrhoeagenic *Escherichia coli*, including enteroaggregative *E. coli* (EAEC), enteropathogenic *E. coli* (EPEC), enteroinvasive *E. coli* (EIEC), enterotoxigenic *E. coli* (ETEC), diffusively adherent *E. coli* (DAEC) and Shiga-toxin producing *E. coli* (STEC) (Hien et al., 2008; de Mello Santos et al., 2020). Intestinal parasites such as *Cryptosporidium parvum* and *Cystoisospora belli* (Ogunlesi et al. 2006; Sunnotel et al., 2006) as well as viruses including norovirus, adenoviruses and rotaviruses have also been implicated as agents of diarrhoea (World Gastroenterology Organization, 2012).

Years ago, highly active antiretroviral therapy (HAART) was not readily available for and accessible to many people living with HIV leading to high mortality rates, but the trend has changed since access to HAART has improved (Ogunbosi et al., 2011). Moreover, Prevention of Mother-to-Child Transmission (PMTCT) programmes have achieved some success averting several potential paediatric HIV infections. In Nigeria, Olakunde et al. (2019) reported 89.5% increase in PMTCT coverage from 2009 to 2016. All young children are vulnerable to diarrhoea but it is possible that children living with HIV, who may be more significantly impacted by diarrhoea sequelae, are infected with a wider repertoire of pathogens and/or experience more severe infections (Tiruneh et al., 2022). Among indicators of diarrhoeal disease severity are the presence of occult blood in the stools, which may also be connected to colorectal cancer and other forms of carcinomas (Antoniou et al., 2015) but is sparsely investigated in the context of diarrhoeal infections. Diarrhoea is the chief predictor of HIV among children but very few African studies have focused on microbial aetiology of this syndrome in children living with HIV (Okeke and Nataro, 2001; Samie et al., 2007; Ogunbosi et al., 2011). Studies focused on the repertoire of diarrhoea aetiologies among people with HIV in Africa are scarce overall with information largely unavailable in Nigeria, and no information on aetiologies since HAART access improved. This study investigates the facultative aerobic bacterial and parasitic (but not viral) aetiologies of diarrhoea, and stool occult blood, in children living with HIV and compares them to aetiology in children without HIV.

## Materials and methods

### Study area and patient enrolment

The study was conducted in two healthcare facilities, the University College Hospital (UCH) and Adeoyo Maternity Teaching Hospital (AMTH), both in Ibadan, Nigeria. The research was approved by the UI/UCH Ethics Committee with assigned number UI/EC/18/0335 and Oyo State Ethical Review Committee with assigned number AD13/479/1378. Patients’ parents or guardians provided informed consent prior to enrollment.

Children living with HIV and HIV-uninfected controls, aged 0-15 years, were recruited between January 2019 and February 2021. Children Living with HIV were recruited at HIV clinics or wards while HIV-negative controls were recruited at children emergency wards or children outpatient clinics. Recruitments were guided by the national guidelines for screening children for HIV and managing them (Federal Ministry of Health, Abuja, Nigeria, 2016). Briefly, children less than 18 months are screened for HIV using HIV DNA polymerase chain reaction (PCR). Children with positive HIV DNA PCR results are subsequently placed on HAART. Children with negative results get antibody test at 18 months after weaning. For children ≥ 18 months, antibody tests are carried out using Determine™ rapid kit (Abbott Diagnostics, Japan). If reactive, a confirmatory test is done using Stat Pak^®^ (ChemBio Diagnostic Systems, USA). Uni-Gold™ (Trinity Biotech, Ireland) is used as tie breaker in the event of discordant results.

Eligibility criteria for participation in this study include verified HIV status known to and confirmed by the attending medical personnel, passage of loose stools three or more times within a 24-hour period over the past three days and obtained informed consent from their parents or guardians.

### Stool microscopy

Microscopic examination of the stool samples was done using the method of Winn et al. (2006). Drops of normal saline and iodine were separately placed on a clean glass slide and with an applicator stick, an aliquot of stool sample was picked and placed on the drops of normal saline and iodine to make a smear. A cover slip was placed over each wet preparation which was mounted for microscopic observation using the x10 and x40 objectives.

Modified Ziehl-Neelsen method (Fayer et al., 2000; Tahvildar-Biderouni and Salehi, 2014) was also employed to examine the samples for coccidian parasites *Cryptosporidium parvum, Cylospora cayetanensis* and *Cystoisospora belli*. Stool smear was made on clean glass slide and allowed to dry. The dried smear was fixed in methanol for three minutes and subsequently stained with strong carbol fuchsin for 20 minutes and then rinsed in tap water. The smear was counterstained with methylene blue for 60 seconds and rinsed with tap water, then dried. Examination of the slides was done using x40 and x100 objectives.

### Testing for presence of occult blood

Detection of occult blood in the stool samples was done using a rapid kit (Cromatest^®^, Linear Chemicals, Spain). This was done by transferring an aliquot of the stool sample into the specimen collection tube containing the extraction buffer and mixed together by shaking. Subsequently, three drops of the extracted stool sample were transferred to the test cassette and observed for positivity indicated by a red line showing on the region marked “T” (for test) as well as the region marked “C” (control) on the device. Specimens testing negative lacked the red line on the test region but showed the red line on the control region (Yamamoto and Nakama, 2000).

### Bacteriology

The stool samples were cultured on MacConkey agar and Eosin Methylene Blue (EMB) agar for the isolation of *Escherichia coli* and other enteric pathogens. For recovery of *Salmonella* and *Shigella*, the stool samples were first enriched overnight in selenite F broth before being sub-cultured onto xylose lysine deoxycholate (XLD) agar. Incubation was done at 37^0^C for 18-24 hours (Medina et al., 2010). For the detection of *Yersinia* species, stool samples were initially enriched in phosphate buffered saline (PBS) for a 21-day period at 4°C before being sub-cultured onto *Yersinia* selective agar (YSA) and allowed to incubate at room temperature for 24-48 hours. Isolates were purified *via* sub-culturing and cryopreserved in 50:50 Luria Broth-glycerol mixture.

Isolate identification was done by conventional biochemical tests and confirmation using the Vitek2 Identification system (Biomerieux Inc., Hazelwood, MO, USA) that allowed suspensions of the isolates in cassettes to undergo a 64-well reagent test for identification. *Salmonella* spp and diarrhoeagenic *E. coli* were confirmed/differentiated by PCR.

### DNA extraction and PCR

The DNA of isolates extracted using the Promega^®^ wizard genomic extraction kit was used to template PCR employing primers for the detection of virulence genes. The genes sought delineating different pathotypes of diarrhoeagenic *E. coli* and *Salmonella* spp. listed in **Table 1**. Strains were deemed to belong to the respective pathotype if they contained at least one of the gene targets sought for that pathogenic type.

**Table 1:** Primer information of target genes.

Gene target	Orientation	Oligonucleotide sequence (5’→3’)	Amplicon size (bp)	Reference
*aap*	Forward	CTT GGG TAT CAG CCT GAA TG	310	Cerna et al. (2003)
	Reverse	AAC CCA TTC GGT TAG AGC AC		
*aggR*	Forward	CTA ATT GTA CAA TCG ATG TA	457	Cerna et al. (2003)
	Reverse	AGA GTC CAT CTC TTT GAT AAG		
*aatA*	Forward	CTG GCG AAA GAC TGT ATC AT	629	Cerna et al. (2003)
	Reverse	CAA TGT ATA GAA ATC CGC TGT T		
*eae*	Forward	CTGAACGGCGATTACGCGAA	917	Aranda et al. (2007)
	Reverse	CGAGACGATACGATCCAG		
*bfpA*	Forward	AATGGTGCTTGCGCTTGCTGC	326	Aranda et al. (2007)
	Reverse	GCCGCTTTATCCAACCTGGTA		
*elt*	Forward	GGCGACAGATTATACCGTGC	450	Aranda et al. (2007)
	Reverse	CGGTCTCTATATTCCCTGTT		
*est*	Forward	ATTTTTMTTTCTGTATTRTCTT	190	Aranda et al. (2007)
	Reverse	CACCCGGTACARGCAGGATT		
*ipaH*	Forward	GTTCCTTGACCGCCTTTCCGATACCGTC	600	Aranda et al. (2004)
	Reverse	GCCGGTCAGCCACCCTCTGAGAGTAC		
*stx1*	Forward Reverse	ATAAATCGCCATTCGTTGACTAC AGAACGCCCACTGAGATCATC	244	Aranda et al. (2004)
*stx2*	Forward	GGCATGTCTGAAACTGCTCC	190	Aranda et al. (2004)
	Reverse	TCGCCAGTTATCTGACATTCTG		
*invA*	Forward	GTGAAATTATCGCCACGTTCGGCCAA	284	Aranda et al. (2004)
	Reverse	TCATCGCACACGTCAAAGGAACC		

### Data analysis

Data obtained in this study were analyzed using Chi square with p set at 0.05. In the many instances where expected values were <5, the Fisher’s Exact test was used.

## Results and discussion

The United Nations International Children’s Emergency Fund (UNICEF, 2006) report observed that 90% of new cases of HIV among children are acquired through transmission from mother to child. Since then, very few studies have examined the aetiology of diarrhoea in children living with HIV. This study aimed to identify diarrhoeal pathogens in children living with HIV. We enrolled fewer individuals than anticipated when the study was designed five years ago because the target population is on the decline due to improved access to antiretroviral therapy. Olakunde et al. (2019) reported a significant increase in coverage of prevention of mother-to-child transmission (PMTCT) in Nigeria, accounting for the low recruitment of diarrhoeic children living with HIV that limits our analyses. However, the small data we have will support their management and remains relevant as the goal to end paediatric AIDS within the current decade is as yet unmet (Bagcchi, 2022). This study screened for bacterial and parasitic aetiologies of diarrhoea in children living with HIV and HIV-negative controls. Viral agents were unfortunately not sought owing to non-availability of required resources, a second limitation of this study.

### Enteric pathogen detection in diarrhoea stools and co-infections

In this study, only ten children living with HIV who had diarrhoea could be recruited during the study’s 25-month duration and 55 HIV-uninfected children with diarrhoea were recruited as controls. EAEC, EIEC*, C. parvum* and *C. cayetanensis* were the most frequently encountered pathogens in the study subjects, while STEC, *Shigella* and *Yersinia* were not detected at all. The full and repertoire of bacterial and parasitic pathogens detected/isolated from them are presented on **Table 2**.

**Table 2:** Detection of pathogens in the stools of children living with HIV and HIV-uninfected children with diarrhea.

Pathogen	Children living with HIV (n = 10)	HIV- uninfected children (n = 55) (%)
Enteroaggregative *Escherichia coli* (EAEC)	4	14 (25.5)
Enteropathogenic *Escherichia coli* (EPEC)	1	5 (9.1)
Enterotoxigenic *Escherichia coli* (ETEC)	1	4 (7.3)
Enteroinvasive *Escherichia coli* (EIEC)	2	8 (14.5)
Shiga toxin-producing *E. coli* (STEC)	0	0 (0.0)
*Shigella*	0	0 (0.0)
*Yersinia* spp	0	0 (0.0)
*Salmonella* spp	2	2 (3.6)
Cryptosporidium parvum*	4	4 (7.3)
*Cyclospora cayetanensis*	3	4 (7.3)
*Cystoisospora belli*	1	3 (5.5)
*Ascaris lumbricoides*	0	1 (1.8)
*Entamoeba histolytica*	0	1 (1.8)

*Cryptosporidium parvum was recovered more often from stools of children living with HIV than from HIV-negative children (p = 0.02).

**Figure 1:**
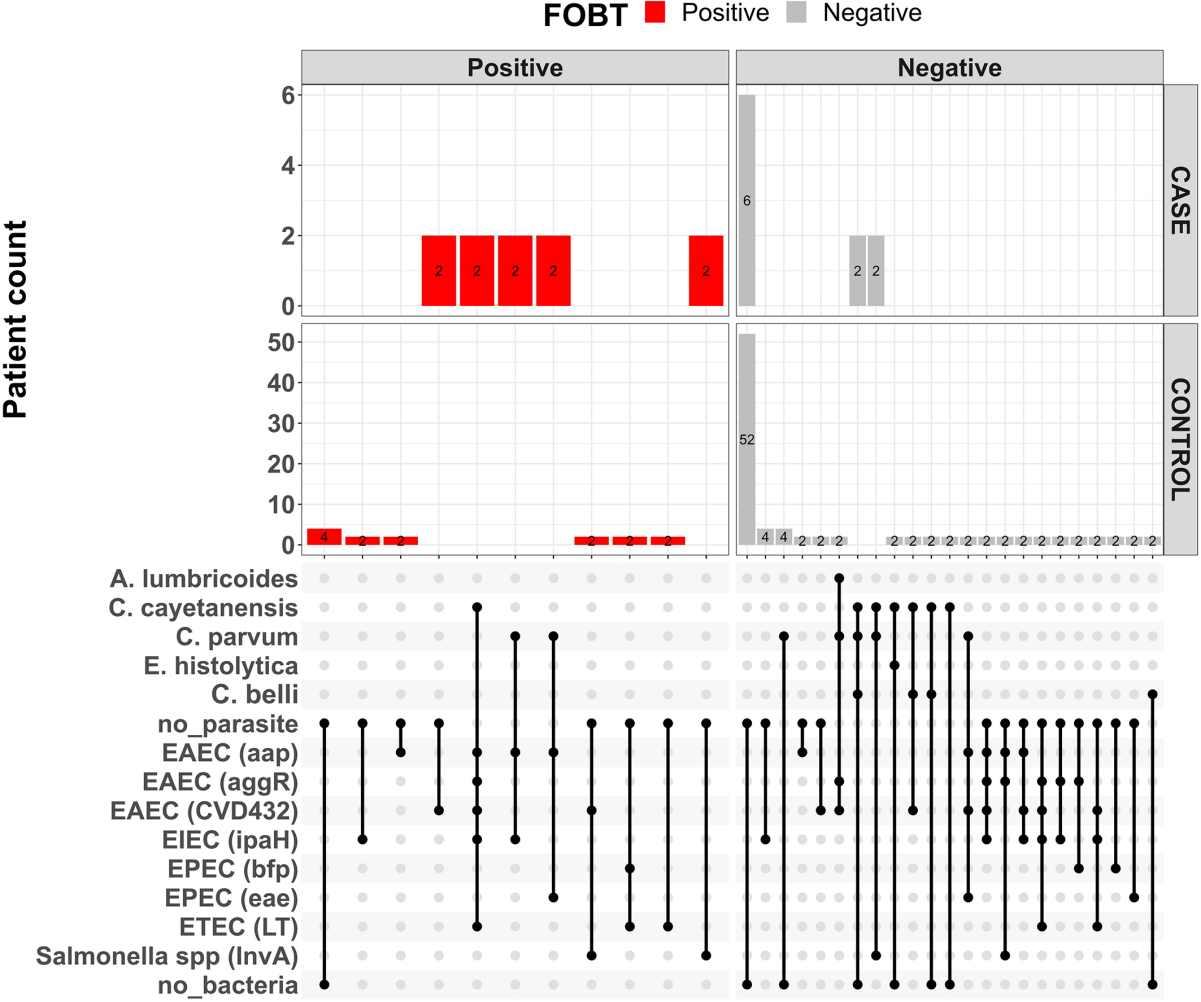
Upsett plot showing pathogen combinations for co-infections and faecal occult blood status among children living with HIV (cases) and HIV-uninfected children (controls).

Other aetiologic studies performed in Ibadan and other locations in Nigeria have found diarrhoeagenic *E. coli*, *Cryptosporidium parvum* and *Cystoisospora belli* commonly associated with diarrhoea (Adesiji et al., 2007; Abdullahi et al., 2010; Saka et al., 2019; Karshima and Karshima, 2021; Akinlabi et al., 2022). These pathogens were all detected in stools from both children living with HIV and the of HIV- uninfected children who served as controls in study (**Table 2**) with comparable recovery rates seen in both patient groups for almost all pathogens. An important exception is *C. parvum*, which was recovered from four of ten children living with HIV but only 4 (7.3%) HIV- uninfected children (p = 0.02, **Table 2**). *C. parvum*, is a major protozoan parasite commonly detected in immunocompromised individuals, particularly people living with HIV, including those in Nigeria (Botero-Garces et al., 2021; Karshima and Karshima, 2021). A meta-analysis of 131 studies by Wang et al. (2018) revealed low prevalence of *Cryptosporidium* (and *Cystoisospora*) among People Living with HIV in high income studies and a much higher prevalence, with diarrhoea association in sub-Saharan Africa. Although we found lower frequencies, *Cyclospora cayetanensis* is also commonly reported from people living with HIV (Stark et al., 2009; Feasey et al., 2011) and *Cystoisospora belli* is increasingly being recognized as a cause of diarrhoea in this sub-population (Casmo et al., 2018). As shown in **Figure 1** at least one parasite was detected in 5/10 of children living with HIV but only 9/55 (16.4%) of children without HIV infection (p = 0.03) showing that parasite detection is associated with HIV.

EAEC was the most frequently detected pathogen in diarrhoeal stools among both children living with HIV and HIV-uninfected children. Reports from within Nigeria (Onanuga et al., 2014; Saka et al., 2019; Akinlabi et al., 2022) and elsewhere (Kotloff et al., 2013; Shah et al., 2016) show that, in the relatively infrequent instances where it is sought, EAEC is commonly the most frequently detected diarrhoeagenic *E. coli* pathotype in children with diarrhoea although it can also be recovered from children without diarrhoea (Franç et al., 2013; Platts-Mills et al., 2015). Pathogen and host factors may be important determinants of virulence and children without diarrhoea who carry EAEC may be at risk of nutrient malabsorption and other less visible sequelae (Nataro et al., 1992; Nataro et al., 1998; Platts-Mills et al., 2015).

Certain enteric bacterial pathogens, notably *Shigella* and ETEC have been prioritized for vaccine development. ETEC strains were recovered from children with and without HIV infection in this study, whereas, *Shigella*, though commonly reported as a pathogen of significance in Africa, was not found in this study (Breurec et al., 2018). While the study size was small, EIEC, which carry the *Shigella* invasive plasmid were recovered from children with and without HIV. Cabal et al. (2016) reported EIEC association with diarrhoea in patients from Spain. Meanwhile, recent studies in Nigeria have not recovered *Shigella* or reported relatively low recovery (Joseph et al., 2017; Akinwumi et al., 2021), adding to evidence supporting earlier speculation that this pathogen may be less important in this setting and therefore the importance of understanding pathogen epidemiologies in a range of settings (Lindsay et al., 2015; Okeke et al., 2016).

Overall, the genes delineating EAEC (*aap. aggR* and *aatA*) were the most commonly detected by PCR, further confirming the preponderance of EAEC among diarrhoeic children in our setting as previously reported by Saka et al. (2019) and Akinlabi et al. (2022) and elsewhere (Weintraub, 2007). In contrast to EAEC, EPEC and *Salmonella* spp. were less commonly recovered with other bacterial pathogens. Of the six (6) EPEC strains recovered, including the one isolate from a child living with HIV, three (3) were typical, that is had the EPEC adherence factor plasmid-borne *bfpA* gene, and three (3) were atypical, that is lacked the gene

Multiple pathogens were detected among five of the ten children living with HIV (**Table 3**), representing half of the children in the group whereas multiple detection of pathogens was observed in 14 (25.5%) of the 55 HIV-uninfected children. Ledwaba et al. (2018) reported bacteria-bacteria co-infections among diarrhoiec children of rural communities of Vhembe district in South Africa, an observation also made in the current study. In a study done in Uruguay (Varela et al., 2015), mixed infections were observed only in 2 (3.4%) of the diarrhoea patients. Unsurprisingly in this study, there is preponderance of EAEC in the mixed infections, as it was the most frequently encountered pathogen overall. However, certain combinations were encountered more frequently than would be predicted by chance alone. For example, as shown in **Table 2**, EAEC and EIEC were seen in combination in three of the ten HIV-uninfected children, whereas probabilities predict that 0-1 children would be expected to carry both pathogens by chance alone based on overall detection rate. Similarly, the combinations of EAEC and *Salmonella* spp. as well as EAEC, EIEC and ETEC were each detected in two HIV-uninfected children with expected carriage rates of ¾1 child respectively. The detection of multiple pathogens (including combinations of bacterial and parasitic agents, that is bacterial-parasite co-infections) in some of the subjects in this study underscores the multifactorial nature of diarrhoea as was previously reported by Kotloff et al. (2013) in the Global Enteric Multicentre Study (GEMS). Zhang et al. (2016) also reported enteric pathogens co-infections among diarrhoeic children in China. Chattaway et al. (2013) reported common co-infections of EAEC with other pathogens albeit being more significantly recovered from diarrhoeic cases than healthy controls.

**Table 3:** Pathogens identified in diarrhoeal stool specimens from the ten children with HIV.

Subject code	Parasites detected	Bacteria isolated	Number of times specific combination was seen in HIV- negative children with diarrhoea (n=55)
AC01X	none	none	NA
NC01X	none	none	NA
NC02X	none	EAEC	NA
NC03X	none	*Salmonella* spp	NA
NC04X	none	none	NA
**NC05X**	*Cryptosporidium parvum*	EAEC, EPEC	1
**NC06X**	*Cryptosporidium parvum, Cyclospora cayetanensis, Cystoisospora belli*	none	0
**NC07X**	*Cryptosporidium parvum, Cyclospora cayetanensis*	*Salmonella* spp	0
**NC08X**	*Cryptosporidium parvum*	EAEC, EIEC	0
**NC09X**	*Cyclospora cayetanensis*	EAEC, EIEC, ETEC	0

NA, Not applicable.

Subjects from whom multiple pathogens were recovered are marked in bold font.

**Figure 1** shows a plot of the pathogen combinations observed in this study. The repertoire of pathogens among the children living with HIV and HIV-uninfected children appear similar. However, the proportion of children living with HIV who had either parasites or bacteria detected/recovered from their stools was higher than HIV-uninfected children. The detection of similar repertoire of diarrhoeagenic *E. coli* pathogens from HIV-infected and HIV-uninfected children has previously been reported by Samie et al. (2007). Even though viruses are part of the leading causes of childhood diarrhea (Nguyen et al., 2007), they were not sought in this study and therefore it is possible that more infections might have been mixed than we are able to report.

### Occult blood in stool

Gross or occult (hidden) in faeces can have different origins, ranging from injury to the epithelium and mucosal linings of the gastrointestinal tract by invasive pathogens (Bardhan et al., 2000) to colorectal cancer (CRC) (Borges et al., 2018). Blood in the stool delineates acute watery diarrhoea from dysentery, which is important since the two syndromes are managed differently and understanding which pathogens produce either or both syndromes is important for understanding the pathogenesis of diarrhoeal pathogens in hosts of differing susceptibilities. When blood in stool is not macroscopically visible, that is occult, it has to be detected through tests for blood components in stool, which are generally reliable although they may also pick up heme components that have been ingested with food (Rose et al., 1990; Ransohoff and Lang, 1997; Craven, 2001). For infectious diarrhoea, occult blood is a marker of severe disease caused by invasive pathogens such as *Shigella, Salmonella* and *Entamoeba histolytica sensu stricto*. Gassama-Sow et al. (2004) had reported high levels of severe diarrhoea among people living with HIV.

In this study, fecal occult blood detection was not associated with number and nature of pathogen recovered but showed significant association with HIV positive status (p = 0.01) as shown in **Table 4**. Bacterial pathogens were recovered from 6 of the children living with HIV, 5 of who had occult blood detected in their stool samples whereas of the 20 HIV-negative children from whom bacterial pathogens were isolated, only 5 had their stool samples positive for occult blood (p = 0.02). Similarly, 3 of the 5 parasite-positive children living with HIV also had their stool samples positive for occult blood compared to none (0) of the 9 parasite-positive children without HIV infection in the control group (p = 0.003). Barring earlier stated confounders such as possibility of colorectal cancer and dietary influences among recruited subjects, our data suggest that invasive pathogens may exert the worst toll on children living with HIV.

**Table 4:** Markers of severity: Multiple pathogens and occult blood in stool specimens from children with diarrhoea.

Variable	Children living with HIV (n = 10)	HIV-uninfected children (n = 55) (%)	p value
Bacteria-parasite coinfection	3	3 (5.5)	0.04*
Multiple pathogens	5	12 (21.8)	0.11
Fecal occult blood positivity	5	7 (12.7)	0.01*

A limitation of this study, in addition to the unavoidably small sample size, is that it did not investigate other pathogens such as *Campylobacter* species and viruses that have been implicated in diarrhoea aetiology (Junaid et al., 2011; Imade and Eghafona, 2015). Even though the study focused on a limited number of bacterial and parasitic etiologies of diarrhoea, multiple pathogens in form of bacteria-bacteria, bacteria-parasite and parasite-parasite co-infections were observed in the specimens. Meanwhile, the overall breadth of pathogens detected in specimens from children living with HIV compares with that of the HIV-uninfected children (**Table 4**). However, bacteria-parasite co-infection in specimens from children living with HIV when compared to those from children without HIV infection (p = 0.004).

## Conclusion

A broad range of diarrhoeal pathogens are detectable in the stools of children living with HIV that present with diarrhoea in Ibadan and the pathogen repertoire is similar to what is seen with HIV-uninfected children with diarrhoea. Children living with HIV who have diarrhoea often present with blood in the stool suggesting that their infections could be invasive and therefore more severe. Mixed infections are common, particularly among children living with HIV so that general Water, Sanitation and Hygiene (WASH)-based interventions are most likely to have significant protective effects.

## Data availability statement

The original contributions presented in the study are included in the article/supplementary material. Further inquiries can be directed to the corresponding author.

## Ethics statement

The studies involving human participants were reviewed and approved by University of Ibadan/University College Hospital Ethics Committee and Oyo State Ethical Review Committee. Written informed consent to participate in this study was provided by the participants’ legal guardian/next of kin.

## Author contributions

OB: conceptualization, data collection, formal analysis, investigation, methodology, project administration, and visualization, writing first draft. MO: investigation, data collection. BO. conceptualization, investigation, methodology, supervision and resources. OA: conceptualization, investigation, methodology, supervision and resources KA: conceptualization, investigation, methodology, supervision and resources TI: conceptualization, investigation, methodology, supervision and resources VO investigation, methodology, supervision VN: investigation AG-A investigation, data collection EA: investigation, data collection IO: conceptualization, formal analysis, funding acquisition, investigation, methodology, project administration, supervision, resources and visualization. All authors contributed to the article and approved the submitted version.
